# A comparison of simplified versus the original media in standard vapor freezing and in-house vitrification of human sperm

**DOI:** 10.5935/1518-0557.20250154

**Published:** 2026

**Authors:** Natpat Jansaka, Ubol Saeng-anan, Waraporn Piromlertamorn, Teraporn Vutyavanich, Usanee Samee

**Affiliations:** 1 Division of Reproductive Medicine, Department of Obstetrics and Gynecology, Faculty of Medicine, Chiang Mai University, Chiang Mai 50200, Thailand; 2 CMEx Fertility Center, Center of Medical Excellence, Chiang Mai University, Chiang Mai 50200, Thailand

**Keywords:** simplified medium, sperm cryopreservation, vitrification, vapor freezing

## Abstract

**Objective:**

We compared post-cryopreserved outcomes of normozoospermic semen samples after cryopreservation by vitrification and liquid nitrogen vapor freezing, using the original and the simplified preservation media.

**Methods:**

Forty normozoospermic semen samples were used in the study. Post-prepared semen samples were divided into five aliquots: one served as non-cryopreserved control; two were vitrified using in-house (In-house-V) or sucrose media (Simp-V); and the last two aliquots were frozen in liquid nitrogen vapor, using commercial (Com-L) or sucrose media (Simp-L).

**Results:**

Sperm after cryopreservation regardless of the media and method used, significantly decreased in motility, viability, and increased ROS level without changes in sperm morphology and DNA fragmentation. Simplified sucrose freezing medium significantly increases post-thawed motility (57.6% (53.2-68.9) vs. 34.5% (27.3-43.6) in Simp-L and Com-L) and viability (61.0% (52.0-67.8) vs. 37.0% (29.3-46.0) in Simp-L and Com-L) in vapor freezing but significantly decrease post-thawed motility (58.8% (51.4-63.3) vs. 77.8% (70.8-81.3) in Simp-V and In-house-V) and viability (59.0% (53.2-66.0) vs. 75.5% (68.0-83.0) in Simp-V and In-house-V) in vitrification. A simplified medium does not affect sperm morphology, ROS level, and DNA fragmentation.

**Conclusions:**

In liquid nitrogen vapor freezing, a simplified medium significantly improved sperm motility and viability compared with commercial medium. In vitrification, the simplified medium gave inferior results on sperm motility and viability compared to the original preservation medium.

## INTRODUCTION

Human sperm cryopreservation is currently an important technique in assisted reproductive technology. There are several sperm cryopreservation techniques and cryoprotective agents (CPAs) available (Agha-Rahimi *et al.*, 2014). CPAs are added before freezing to prevent cryodamage. Several CPAs have been developed and used successfully. CPAs exert their effects via various mechanisms, such as decreasing the freezing point of water and producing membrane protection. Normally, CPAs are categorized into permeable and non-permeable groups. Permeable CPAs are used for routine slow sperm cryopreservation, but high concentrations of these agents show toxicity and decrease sperm fertility potential (Gilmore *et al.*, 1997; Hoffmann *et al.*, 2011). Traditionally, glycerol is the main CPA for sperm freezing but it is considered to be a toxic compound (Vidament *et al.*, 2009).

Liquid nitrogen vapor freezing may be classified as a type of rapid freezing. The mixed sample of spermatozoa and CPAs is loaded into a straw or cryovial and incubated at room temperature. Then, the mixture is exposed to the liquid nitrogen vapor phase at -80°C for about 10 minutes before plunging into liquid nitrogen (Di Santo *et al.*, 2012). A study comparing vapor freezing and slow programmable freezing reported a significantly greater rate of chromatin deterioration with rapid cooling than with slow freezing (Hammadeh *et al.*, 2001). Vitrification is the process of cooling cells at an extremely high rate. With vitrification, liquid turns into a glass-like solidification state, without ice crystal formation. Vutyavanich *et al.* (2010; 2012) proposed improvement in sperm vitrification through a solid surface vitrification (SSV) system. The cryopreservation medium was modified by reducing the concentration of glycerol from 15% to 10% to minimize CPA toxicity. The results showed significantly improved sperm motility and survival, without change in normal sperm morphology and DNA fragmentation, when compared with the conventional slow programmable freezing. One study showed a significant increase in DNA fragmentation following SSV and rapid freezing techniques (Satirapod *et al.*, 2012), but Vutyavanich *et al.* (2010; 2012) and Isachenko *et al.* (2004) observed no significant differences in DNA integrity following vitrification or rapid freezing techniques.

As spermatozoa have scanty, if any, cytoplasm remaining with much sensitivity to permeable CPA. A research question is raised whether sperm cryopreservation outcomes can be improved by omitting permeable CPAs altogether. Woelders (1997) reported a slight improvement in sperm cryopreservation when sucrose rather than trehalose was used as a non-permeable CPA. On the other hand, another study demonstrated a higher glass transition temperature for trehalose than sucrose, implying a better vitrification property of trehalose over sucrose (Simperler *et al.*, 2006). Liu *et al.* (2016) confirmed that trehalose and sucrose were the most effective CPAs. The study by Rahiminia *et al.* (2017), using 0.5 M sucrose in sperm vitrification, reported good recovery of motility, morphology, and viability, while sperm chromatin and acrosome integrity remained unaffected.

The objective of this study was to determine whether the simplified cryopreservation medium, containing only sucrose, would result in similar post-cryopreserved outcomes when compared with the original media for sperm vitrification and liquid nitrogen vapor freezing. The outcomes measured were sperm motility, morphology, viability, reactive oxygen species (ROS) level, and sperm DNA fragmentation.

## MATERIAL AND METHODS

### Participant selection criteria

Semen samples were obtained from male partners of infertile couples, who visited the CMEx Fertility Center at Maharaj Nakorn Chiang Mai Hospital. The samples were collected into sterile containers by masturbation after a two to seven day abstinence. Only semen with normal parameters, according to the World Health Organization reference values (WHO, 2021) were included in the study. The study was reviewed and approved by the Research Ethics Committee, Faculty of Medicine, Chiang Mai University. All participants gave their written informed consent for the use of their semen for research.

### Experimental design

**Figure 1:**
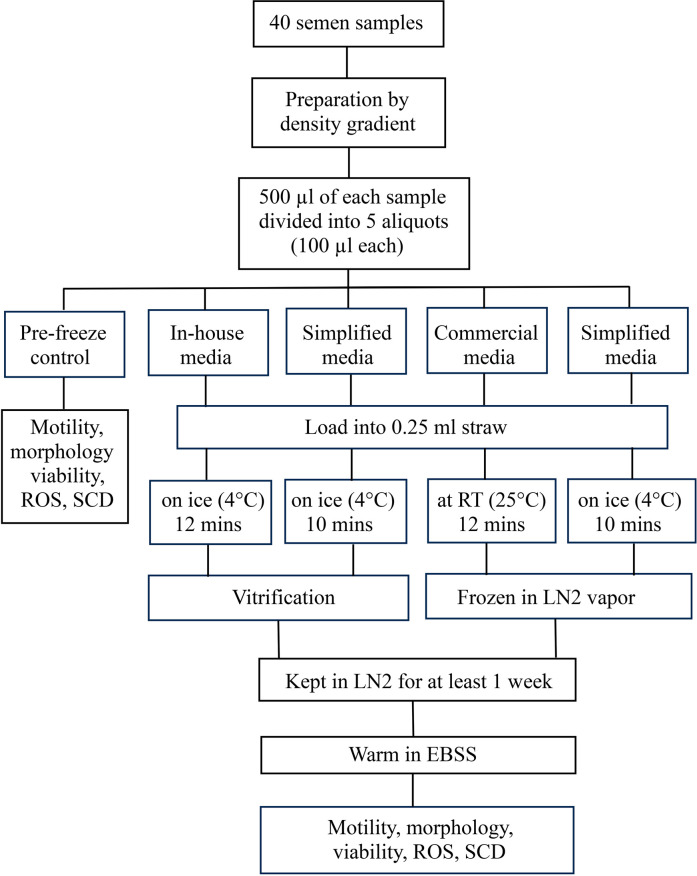
Flow diagram of study. Abbreviation: RT = room temperature, LN2 = liquid nitrogen, ROS = reactive oxygen species, SCD = sperm chromatin dispersion test.

Samples were prepared with the density gradient centrifugation method. Liquefied semen samples were layered on top of 80%/40% discontinuous Sil-Select gradients (FertiPro N.V., Beernem, Belgium) and centrifuged at 350 *g* for ten minutes at 25°C. After that, the sperm pellet was washed twice in sperm washing medium, supplemented with 0.3% (w/v) human serum albumin (HSA; LifeGlobal, Guilford, CT), at 300 *g* for five minutes. The supernatant was discarded, and the final pellet was resuspended in 500 µL of the same medium and divided into five aliquots. The first 100 µL aliquot served as a non-frozen control and was immediately assessed for sperm motility, kinetics, morphology, viability, ROS levels, and DNA integrity. The remaining four aliquots were cryopreserved by vitrification or liquid nitrogen vapor freezing using the original media for sperm vitrification and liquid nitrogen vapor freezing or the simplified media, containing only sucrose. The flow of the study is shown in Figure 1.

### Sperm cryopreservation media

The original media for sperm vitrification using the in-house made medium contained 10% glycerol, 10% HSA, 133 mM glycine, 5.5 mM glucose, 100 mM Trehalose, 12.2 mM sodium pyruvate, and 20 mM HEPES. The in-house medium was a modified human sperm medium by reducing the concentration of glycerol from 15% to 10% to minimize CPA toxicity. More viscosity was created by increasing the HSA 20-fold and replacing 50 mM of sucrose with 100 mM of trehalose (Vutyavanich *et al.*, 2010). The original media for liquid nitrogen vapor freezing using commercial media (Sperm Freezing, Lifeglobal, USA). The simplified medium consisted of 0.5 M of sucrose, HSA (10 g/L), and phosphate-buffered saline (PBS).

### Vitrification and warming

Vitrification was performed on the second and third aliquots. The aliquots, containing 100 µl of prepared semen, were mixed dropwise with an equal volume of cryoprotective medium. The second aliquot was mixed with the original in-house medium, while the third aliquot was mixed with the simplified sucrose medium. The mixtures were loaded into 0.25 mL straws and incubated at 4°C for 12 minutes for the second aliquot and 10 minutes for the third aliquot. The straws were then inserted into the holes of a pre-cooled in-house made aluminum block, previously immersed in liquid nitrogen for 10 minutes (Vutyavanich *et al.*, 2010). For warming, the straws were warmed with water at room temperature (25-28˚C). The samples were then washed with EBSS, and centrifuged at 200 g for five minutes to remove cryoprotective agents. Post-warmed samples were immediately assessed for sperm motility, kinetics, morphology, viability, ROS level, and DNA integrity.

### Liquid nitrogen vapor freezing and thawing

Liquid nitrogen vapor freezing was performed on the fourth and fifth aliquots. The aliquots, containing 100 µl of prepared semen, were mixed dropwise with an equal volume of cryoprotective medium. The fourth aliquot was mixed with the original commercial sperm freezing medium, then loaded into 0.25 mL straws and left to incubate at room temperature for 12 minutes according to the manufacturer’s recommendation. The fifth aliquot was mixed with the simplified sucrose medium. The mixtures were loaded into 0.25 mL straws and left to incubate at 4°C for 10 minutes as in the study of Rahiminia *et al.* (2017). After incubation, straws were placed horizontally at 10 cm above liquid nitrogen level for 12 minutes. For thawing, the straws were placed in warm (37˚C) water. The samples were then washed in EBSS and immediately assessed for sperm motility, kinetics, morphology, viability, ROS level, and DNA integrity.

### Sperm assessment

Sperm motility and kinetics were assessed using an HTM IVOS II computer-assisted semen analyser (CASA; Hamilton Thorne Biosciences, Beverly, MA), equipped with Clinical Human Motility II software. The kinematic parameters measured included: the velocity of smooth average cell path (VAP), mean curvilinear velocity (VCL), mean straight-line velocity (VSL), the amplitude of lateral head displacement (ALH), percent linearity (LIN=VSL/VCLx100) and percent straightness (STR=VSL/VAPx100).

For sperm morphology assessment, the washed samples were smeared on glass slides and labeled accordingly. They were stained with Diff-Quick and assessed with an HTM IVOS II computer-assisted semen analyser (CASA; Hamilton Thorne Biosciences, Beverly, MA). For every slide, at least 200 spermatozoa were read in duplicates.

Sperm viability was assessed by vital staining using 0.5% (w/v) eosin-Y (Sigma Chemical, St. Louis, MO, USA). Ten µL of each aliquot was mixed with 10 µL of 0.5% (w/v) eosin-Y on a glass slide for at least 30 seconds, and 200 spermatozoa were counted as stained (dead) or unstained (viable) under a light microscope.

The ROS level was assessed by a chemiluminescence technique, using a Glomax 20/20 luminometer (Turner Biosystems Inc., Sunnyvale, CA, USA). In essence, ROS and specific reagents reacted and emitted photons that passed through the photomultiplier tubes of the luminometer. The results were measured as relative light units (RLU) of counted photons per minute (CPM) or mV/s. The reagent was prepared using 20 µl of luminol stock solution (5-amino-2,3-dihydro-1,4-phthalazinedione, Cat. No A8511; Sigma Chemical) mixed with 380 µl of DMSO (Cat. No. D8779; Sigma Chemical) in a foil-covered polystyrene tube. The positive control was a mixture of 400 µl of phosphate-buffered saline (PBS), 50 µl of hydrogen peroxide and 10 µl of luminol reagent. The negative control was a mixture of 400 µl of PBS and 10 µl of luminol reagent. Both positive and negative controls were prepared immediately before use. Four-hundred µl PBS was used to dilute 20 µl of a semen sample from each aliquot, and mixed with 10 µl of luminol reagent. Each sample, including the positive and negative controls, was measured twice, and the crude average value of RLU/sec was corrected by dividing it with the sperm concentration to give the final value of ROS expressed in the unit of RLU/sec/10^6^.

The sperm DNA integrity was assessed by a sperm chromatin dispersion (SCD) test.

The SCD test is based on the principle that embedded sperms in an agarose matrix on a glass slide, following acid denaturation and removal of nuclear proteins. Sperm with DNA fragmentation cannot produce the halo of dispersed DNA loops, whereas the dispersed sperm chromatin is present if DNA is not fragmented (Fernández *et al.*, 2003). The level of DNA fragmentation of sperm was evaluated by DNA Fragmentation Software. We chose this test because of its simplicity, reproducibility, and availability without the need for complex or costly instrumentation (De Jonge, 2002). Moreover, high correlations were found between the SCD test and sperm chromatin structure assay (r=0.71; *p*<0.001) and between the SCD test and modern TUNEL assay (r=0.70; *p*<0.001) (Ribas-Maynou *et al.*, 2013).

### Statistical analysis

The IBM SPSS Statistics (Version 21.0, Armonk, NY, USA) was used for statistical analysis. The distributions of the variable data were tested with the Kolmogorov-Smirnov test of normality. Mean age, sperm parameters, and normal distribution data were expressed as mean±SD. Comparison of these variables between the groups was accomplished by repeated measure analysis of variance (ANOVA), When there was a significant difference. Bonferroni post hoc tests were performed. On the other hand, abnormal distribution data was expressed as median (interquartile range). Comparison of these variables between the groups was assessed with the use of Friedman’s test, and then pairwise comparisons for the subgroup variables were observed. A *p*-value of*<*0.05 was considered to be statistically significant.

## RESULTS

Forty normozoospermic semen samples were included in this study. Patients’ age and pre-processing sperm parameters are shown in Table 1. Sperm after cryopreservation regardless of the media and method used, demonstrated a significant decrease in motility and viability, and significantly increased ROS levels. Sperm morphology and DNA fragmentation were comparable in post-cryopreserved sperm and the control (Table 2). A significant decrease in VAP, VSL, VCL and ALH after sperm cryopreservation in both vitrification and liquid nitrogen vapor freezing compared to pre-freeze control. BCF increased significantly and LIN decreased significantly in liquid nitrogen vapor freezing compared to the control (Table 3).

**Table 1:** Age and sperm parameters.

Parameters	Mean±SD
Age (years)	31.5±4.9
Volume (mL)	2.9±1.3
pH	7.9±0.3
Sperm concentration (million/mL)	89.4±59.2
Total motility (%)	69.5±12.2
Progressive motility (%)	62.9±12.9
Normal morphology (%)	8.1±3.4

**Table 2:** Sperm motility, viability, morphology, ROS levels, and DNA integrity in controls and post-cryopreserved samples in different media and methods.

Parameters (n=40)	Control	Vitrification		Liquid nitrogen vapor	
		In-house-V	Simp-V	Com-L	Simp-L
Total motility (%)	92.4 (85.4-95.3)^*^	77.8 (70.8-81.3)	58.8 (51.4-63.3)^†^	34.5 (27.5-43.6)	57.6 (53.2-68.9)^‡^
Progressive motility (%)	89.2 (84.5-93.0)^*^	67.5 (60.0-72.0)	49.0 (41.1-55.3)^†^	28.5 (21.7-34.9)	50.6 (42.9-56.3)^‡^
Viability (%)	90.5 (86.0-93.0)^*^	75.5 (68.0-83.0)	59.0 (53.3-66.0)^†^	37.0 (29.3-46.0)	61.0 (52.0-67.8)^‡^
Normal morphology (%)	14.9 (8.3-20.5)	12.3 (5.3-16.2)	12.0 (9.0-16.9)	11.1 (7.4-16.3)	12.4 (7.6-16.6)
ROS (RLU/sec/106)	4.5 (2.3-8.2)^§^	7.4 (2.3-16.5)	8.7 (3.4-15.6)	11.7 (5.9-18.7)	8.1 (4.2-13.4)
DNA fragmentation (%)	24.5 (14.3-30.0)	25.5 (19.3-32.0)	27.5 (21.3-37.8)	30.0 (20.0-36.0)	26.5 (23.0-29.0)

Data expressed as median (interquartile range)

Friedman’s test,

* *p*<0.001 *vs*. all groups (In-house-V, Simp-V, Com-L, Simp-L),

† *p*<0.001 *vs*. In-house-V,

‡ *p*<0.001 *vs*. Com-L,

§ *p*<0.05 *vs*. all groups (In-house-V, Simp-V, Com-L, Simp-L)

Abbreviation: In-house = in-house media, Simp = simplified sucrose media, Com = commercial media, V = vitrification, L = liquid nitrogen vapor freezing, ROS = reactive oxygen species.

**Table 3:** Sperm kinemetics in controls and post-cryopreserved samples in different media and methods.

Parameters (n=40)	Control	Vitrification		Liquid nitrogen vapor	
		In-house-V	Simp-V	Com-L	Simp-L
VAP (µm/sec)	72.9 (67.0-79.5)^*^	60.4 (55.3-68.3)	57.2 (51.8-60.1)	54.5 (49.5-59.0)	56.7 (53.1-62.5)
VSL (µm/sec)	61.5 (53.3-66.8)^*^	52.5 (46.8-57.3)	48.8 (42.7-51.3)	44.8 (41.1-49.9)	47.8 (43.9-51.9)
VCL (µm/sec)	116.2 (110.3-130.1)^*^	100.6 (92.8-116.7)	97.5 (85.4-109.0)	93.9 (86.5-101.9)	96.2 (88.3-106.0)
ALH (µm)	5.9 (5.5-6.5)^*^	5.0 (4.6-6.1)	5.0 (4.5-5.6)	5.0 (4.4-5.4)	5.0 (4.6-5.6)
BCF (Hz)	25.7 (23.2-27.9)^†^	27.5 (25.8-30.2)	28.0 (26.3-30.9)	32.3 (29.3-36.0)	28.4 (26.9-30.0)^‡^
STR (%)	82.1 (77.7-85.4)	82.8 (80.4-86.3)	81.1 (79.8-84.6)	81.5 (77.8-84.9)	82.6 (78.8-84.1)
LIN (%)	53.2 (47.5-58.4)†	50.2 (46.9-54.2)	48.3 (45.8-52.4)	48.2 (45.3-52.8)	49.2 (45.8-51.9)

Data expressed as median (interquartile range)

Friedman’s test,

* *p*<0.001 *vs*. all groups (In-house-V, Simp-V, Com-L, Simp-L),

† *p*<0.001 *vs*. Simp-V, Com-L and Simp-L,

‡ *p*=0.003 *vs*. Com-L

Abbreviation: In-house = in-house media, Simp = simplified sucrose media, Com = commercial media, V = vitrification, L = liquid nitrogen vapor freezing, VAP = velocity of smooth average path, VSL = mean straight-line velocity, VCL = mean curvilinear velocity, ALH = amplitude of lateral head displacement, BCF = beat cross frequency, STR = percent straightness, LIN = percent linearity

### Simplified media and vitrification

A significant decrease in sperm total motility (58.8% (51.4-63.3) *vs*. 77.8% (70.8-81.3), *p*<0.001), progressive motility (49.0% (41.1-55.3) *vs*. 67.5% (60.0-72.0), *p*<0.001), and viability (59.0% (53.2-66.0) *vs*. 75.5% (68.0-83.0), *p*<0.001) in a simplified media group compared to the original in-house media group (Table 2). There were no differences in sperm morphology (12.0% (9.0-16.9) *vs*. 12.3% (5.3-16.2)), ROS level (8.7 (3.4-15.6) RLU/sec/10^6^ *vs*. 7.4 (2.3-16.5) RLU/sec/10^6^), and DNA fragmentation (27.5% (21.3-37.8) *vs*. 25.5% (19.3-32.0)) in post-vitrified sperm using simplified media or in-house media (Table 2). There were no differences in sperm kinematics after warming in a simplified media group compared to the original in-house media group (Table 3).

### Simplified media and liquid nitrogen vapor freezing

A significant increase in sperm total motility (57.6% (53.2-68.9) *vs*. 34.5% (27.5-43.6), *p*<0.001), progressive motility (50.6% (42.9-56.3) *vs*. 28.5% (21.7-34.9), *p*<0.001), and viability (61.0% (52.0-67.8) *vs*. 37.0% (29.3-46.0), *p*<0.001) in a simplified media group compared to the original commercial media group (Table 2). There were no differences in sperm morphology (12.4% (7.6-16.6) *vs*. 11.1% (7.4-16.3)), ROS level (8.1 (4.2-13.4) RLU/sec/10^6^ *vs*. 11.7 (5.9-18.7) RLU/sec/10^6^), and DNA fragmentation (26.5% (23.0-39.0) *vs*. 30.0% (20.0-36.0)) in post-thawed sperm using simplified media or commercial media (Table 2). BCF decreased significantly in post-thaw sperm using simplified media compared to the commercial media, while other kinematic parameters were not different (Table 3).

## DISCUSSION

Compared to other cells, the spermatozoa are rather unique in that they contain very scanty cytoplasm, as most cytoplasm has been shed during spermiogenesis. Using cryo-scanning electron microscopy and freeze substitution, Morris *et al.* (2007) showed that no intracellular ice was formed inside the sperm during rapid cooling. Currently, it is believed that cryodamage to spermatozoa predominantly result from an osmotic imbalance encountered during thawing rather than the formation of intra-cellular ice (John Morris *et al.*, 2012). Sperm membranes are now under focus as the primary site of cryopreservation injury (Sieme *et al.*, 2015). This revolutionized concept raises the question of whether it is necessary to add any permeable CPA, such as glycerol, into the sperm preservation medium to prevent intracellular ice formation. Moreover, the cost of the simplified medium was lower than the commercial medium. The sucrose containing medium was easily prepared and could be used for vitrification and liquid nitrogen vapor freezing. Indeed, some recent studies showed good results of human sperm vitrification using 0.25-0.5 M sucrose, without the addition of glycerol (Isachenko *et al.*, 2011; Rahiminia *et al.*, 2017).

Our study confirmed previous studies that 0.5 M sucrose, without glycerol, can be used effectively to cryopreserve human sperm (Isachenko *et al.*, 2012; Agha-Rahimi *et al.*, 2014; Chen *et al.*, 2015; Slabbert *et al.*, 2015; Rahiminia *et al.*, 2017). The results were better than those using conventional liquid nitrogen vapor freezing in terms of total and progressive motility, and viability. The percentage of spermatozoa with DNA fragmentation and ROS level were slightly lower than that in the conventional liquid nitrogen vapor freezing, but not statistically significant. Our total and progressive motility of 34.5% and 28.5%, respectively, with conventional vapor freezing was similar to other studies by Satirapod *et al.* (2012), O’Connell *et al.* (2002), and Tiwari *et al.* (2017).

A simplified medium gave significantly inferior outcomes than our original in-house medium for sperm vitrification in sperm motility, progressive motility, and viability. This might have been due to the absence of glycerol and the presence of lower protein concentration in the simplified medium. Glycerol might exert other protective effects on sperm membranes rather than its well-known effect in preventing intracellular ice formation. Indeed, in model studies with liposomes, glycerol has been shown to stabilize membranes and prevent freezing-induced leakage of intraliposomal solutes, whereas non-permeable disaccharides, such as sucrose, do not (Oldenhof *et al.*, 2013). Glycerol and protein have antioxidant activity and could be beneficial in counteracting the increased production of ROS occurring during the cryopreservation. The concentration of glycerol in the cryoprotective medium required for optimal sperm survival is unknown (Hammitt *et al.*, 1988). In animals, a concentration as low as 3.5% can be toxic as it can damage the sperm membrane and cause depolymerization of the actin cytoskeleton (Macías García *et al.*, 2012). Our original vitrification medium and the commercial medium for liquid nitrogen vapor freezing contain 10% and 15% glycerol, respectively. When they were diluted with processed sperm samples in a ratio of 1:1, the final glycerol concentration in the final mixture would be 5% and 7.5%, respectively. In our vitrification system, the exposure was done at 4°C, while that in liquid nitrogen vapor freezing, the equilibration was done at room temperature (20-25°C). In principle, exposure to a CPA at a lower temperature would limit its diffusion and toxicity to cells. We, therefore, believed that the poor results obtained with the liquid nitrogen vapor freezing using the commercial medium might, in part, be due to the toxicity of glycerol. In future studies, we should investigate whether the addition of glycerol at a concentration of 5-7% to the simplified sucrose medium would be beneficial.

Grizard *et al.* (2007) reported that there were changes in sperm kinematics before any impairment of sperm motility. In their study, the affected parameters were the percentage of progressive motile sperm, VAP, and VSL, followed by VCL, whereas ALH was unchanged. In our study, using the same CASA system and analysis setup, we found a similar adverse effect of cryoinjury on sperm motility, VAP, VSL, VCL, and ALH while STR was not affected. Our study found that there was no difference in sperm morphology after cryopreservation. Ozkavukcu *et al.* (2008) proposed that extracellular ice crystal formation might affect sperm morphology. It may be possible that osmolarity could change during the freezing-thawing process caused by the addition and removal of CPAs followed by the water entry and exit. However, a difference of individual resistance to cryopreservation may explain the contrasting results, despite using the same protocol and automatic method (Maroto-Morales *et al.*, 2016).

We found an increase in the levels of extracellular ROS following cryopreservation, which was in line with other studies by Mazzilli *et al.* (1995) and Wang *et al.* (1997). The magnitude of the increase was around double the baseline levels in the control, and there was a statistical difference. It implied that spermatozoa from different individuals differed in their responses to the stress of cryopreservation. We had no information on the levels of intracellular ROS, as we did not measure them in our study.

Hammadeh *et al.* (2001), and Donnelly *et al.* (2000) reported an increase in the percentage of sperm DNA fragmentation after cryopreservation. This was not confirmed by our study of vitrification and liquid nitrogen vapor freezing. However, when we replaced the original media with a simplified sucrose medium, the increase in DNA fragmentation was observed in vitrification, while a decrease in DNA fragmentation with liquid nitrogen vapor freezing, but not statistically significant. This implied that the choice of CPAs and/or the cryopreservation techniques could have a significant impact on the outcome of cryopreservation. Other components in the cryopreservation media, such as pyruvate, might add to the protective effects through their antioxidant or other properties (Zini & Al-Hathal, 2011).

There are several limitations in our study. First, we included only 40 normozoospermic males. The result of our study, therefore, might not be applicable to infertile men in general, especially those with abnormal sperm parameters. Second, the freezing and thawing process might be another variable. In routine practice, the cane did not stay submerged in liquid nitrogen all the time. It was lift up above the level of liquid nitrogen from time to time to remove samples from other patients as required. Third, we did not have information on the fertilizing potential of these cryopreserved spermatozoa, as we did not perform any sperm function tests, such as acrosomal reaction and sperm-zona binding tests.

In conclusion, the use of a simplified medium significantly improved sperm motility and viability compared to the commercial medium in liquid nitrogen vapor freezing. On the contrary, a simplified medium is not suitable for vitrification due to the significantly decreased sperm motility and viability when compared to the original preservation medium.
